# A four-gene signature predicts survival and anti-CTLA4 immunotherapeutic responses based on immune classification of melanoma

**DOI:** 10.1038/s42003-021-01911-x

**Published:** 2021-03-22

**Authors:** Ying Mei, Mei-Ju May Chen, Han Liang, Li Ma

**Affiliations:** 1 Department of Experimental Radiation Oncology, The University of Texas MD Anderson Cancer Center, Houston, TX USA; 2 Department of Bioinformatics and Computational Biology, The University of Texas MD Anderson Cancer Center, Houston, TX USA; 3 Department of Systems Biology, The University of Texas MD Anderson Cancer Center, Houston, TX USA; 4 The University of Texas MD Anderson Cancer Center UTHealth Graduate School of Biomedical Sciences, Houston, TX USA

**Keywords:** Cancer immunotherapy, Computational models

## Abstract

Cutaneous melanoma is the most malignant skin cancer. Biomarkers for stratifying patients at initial diagnosis and informing clinical decisions are highly sought after. Here we classified melanoma patients into three immune subtypes by single-sample gene-set enrichment analysis. We further identified a four-gene tumor immune-relevant (TIR) signature that was significantly associated with the overall survival of melanoma patients in The Cancer Genome Atlas cohort and in an independent validation cohort. Moreover, when applied to melanoma patients treated with the CTLA4 antibody, ipilimumab, the TIR signature could predict the response to ipilimumab and the survival. Notably, the predictive power of the TIR signature was higher than that of other biomarkers. The genes in this signature, *SEL1L3*, *HAPLN3*, *BST2*, and *IFITM1*, may be functionally involved in melanoma progression and immune response. These findings suggest that this four-gene signature has potential use in prognosis, risk assessment, and prediction of anti-CTLA4 response in melanoma patients.

## Abstract (web summary)

Ying Mei et al. identify a four-gene tumor immune-relevant signature that predicts the overall survival of melanoma patients and their response to the CTLA4 antibody ipilimumab. This study suggests a potential utility of this four-gene signature in prognosis, risk assessment, and prediction of anti-CTLA4 response in melanoma patients.

## Introduction

Cutaneous melanoma (hereafter called melanoma), resulting from malignant transformation of melanocytes, is the most aggressive and lethal type of skin cancer. It is the fifth and sixth most common cancer in men and women, respectively, and its incidence keeps increasing in recent years^1^. Current treatment options for melanoma include surgical resection, chemotherapy, radiotherapy, targeted therapy, and immunotherapy^2,3^. Over the past years, immune checkpoint inhibitors (ICIs) have transformed the treatment of melanoma. Cancer immunotherapies with ICIs, such as agents targeting PD-L1, PD-1, or CTLA4, help the immune system to recognize and attack tumor cells. The use of ICIs can prolong the survival of a subset of patients with melanoma. However, the responses to ICIs are variable, with some melanoma patients achieving tumor regression and others showing disease progression^4^. Thus, predictive biomarkers that can distinguish immunotherapeutic responders from non-responders are urgently needed.

The tumor microenvironment (TME), consisting of complex components including tumor cells, immune cells, and stromal cells, is diverse across patients^5^. Compared with many other cancer types, melanomas are characterized by high immunogenicity and are often infiltrated by immune cells^6,7^. High levels of immune cell infiltration are associated with a favorable prognosis in patients with melanoma^8–10^. Highly immune cell-infiltrated tumors, or “hot” tumors, are more likely to respond to ICI-based therapy, whereas tumors with weak immune cell infiltration, or “cold” tumors, are less responsive to ICIs^11^. Hence, an adequate assessment of the TME of melanoma patients will inform treatment options.

Computational algorithms have been developed to quantitate tumor-infiltrating immune cells using marker genes and RNA-sequencing data^12–15^. For instance, by applying multivariate Cox proportional hazards regression to assess gene association with T cell dysfunction phenotype, Jiang et al.^15^ developed a Tumor Immune Dysfunction and Exclusion (TIDE) signature, which could accurately predict cancer immunotherapy response. However, the TIDE signature consists of 770 genes, making it difficult for clinical application. Another recent study^14^ calculated the immune score of melanoma samples by using the ESTIMATE (Estimation of STromal and Immune cells in MAlignant Tumor tissues using Expression data) algorithm, and 25 genes that best correlated with the immune score were identified. However, there are several limitations. First, prognosis-related genes were not taken into consideration. Second, no statistical models were used to avoid overfitting. Third, this 25-gene signature showed no significant association with the response to immune checkpoint blockade therapies^14^.

In the present study, we established a method based on the immune subtype classification of melanoma to identify a tumor immune-relevant (TIR) signature, which could predict the survival outcome as well as the response to anti-CTLA4 treatment in patients with melanoma. Compared with complex gene signatures, this four-gene signature is much more amenable to clinical practice.

## Results

### Classification of cutaneous melanoma based on immunogenomic profiling

**Fig. 1:**
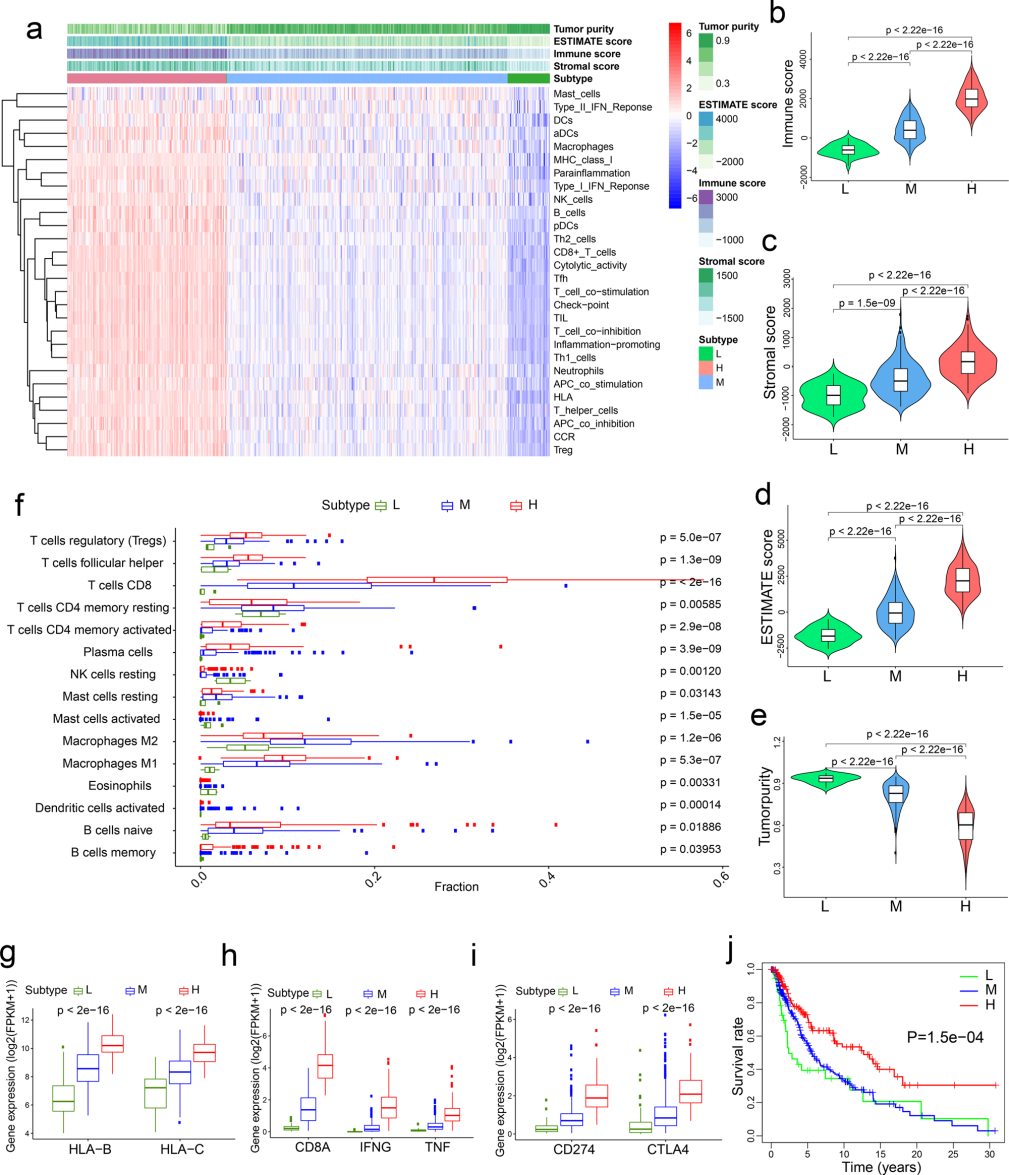
Classification of human skin cutaneous melanoma (SKCM) into three immune subtypes. **a** Hierarchical clustering of TCGA human skin cutaneous melanoma (SKCM) patients (*n* = 471) into three immune subtypes: high-immunity (H), *n* = 156; medium-immunity (M), *n* = 274; and low-immunity (L), *n* = 41. The tumor purity, ESTIMATE score, immune score, and stromal score were determined by the ESTIMATE algorithm. **b**–**e** Comparison of the immune cell infiltration levels (immune score) (**b**), stromal content (stromal score) (**c**), ESTIMATE score (**d**), and tumor purity (**e**) among three immune subtypes of melanoma. **f** Relative abundances of 15 types of immune cells in three immune subtypes of melanoma. **g** mRNA levels of MHC-I genes in three immune subtypes of melanoma. **h** mRNA levels of genes associated with CD8+ T cells in three immune subtypes of melanoma. **i** mRNA levels of PD-L1 and CTLA4 in three immune subtypes of melanoma. The boxplot in **f**–**i** consists of a box and a set of whiskers. The box is drawn from the first quartile (25th percentile) to the third quartile (75th percentile) with a horizontal line drawn in the middle to denote the median. The lowest point is the minimum of the dataset and the highest point is the maximum of the dataset. **j** Kaplan–Meier curves of the overall survival rates in three immune subtypes of melanoma. Statistical significance was determined by the Kruskal–Wallis test in **a**–**i** and by the log-rank test in **j**.

We used the single-sample gene-set enrichment analysis (ssGSEA) algorithm^16^ to quantitate the enrichment levels of 28 immune-associated gene sets in each human skin cutaneous melanoma (SKCM) sample in the TCGA database (*n* = 471). These 28 gene sets represent diverse immune cell types, functions, and signaling pathways^17^ (Supplementary Data 1). Based on the enrichment score of each sample, we performed hierarchical clustering and classified SKCM patients into three immune subtypes: low immunity (the L subtype, *n* = 41), medium immunity (the M subtype, *n* = 274), and high immunity (the H subtype, *n* = 156) (Fig. 1a). The tumor purity, ESTIMATE score, stromal score, and immune score of each sample were calculated using the ESTIMATE algorithm^18^. The immune scores, representing the level of immune cell infiltration, were significantly higher in the H subtype than in the L subtype (*P* < 2.22 × 10^−16^, Fig. 1b). Similarly, the stromal scores, representing the level of stromal content, were significantly higher in the H subtype than in the L subtype (*P* < 2.22 × 10^−16^, Fig. 1c). Tumor purity was calculated based on the ESTIMATE score, which is a composite of the infiltration level of both immune cells and stromal cells. From the L subtype to the H subtype, the ESTIMATE score increased and tumor purity decreased (*P* < 2.22 × 10^−16^, Fig. 1d, e). Collectively, these data suggest that the TME of the high-immunity (H) subtype has high levels of infiltration by immune cells and stromal cells.

### Characteristics of three immune subtypes of melanoma

To validate our finding, we compared the fractions of 15 immune cell types in bulk tumors among the three melanoma subtypes by using the CIBERSORT (Cell-type Identification By Estimating Relative Subsets Of known RNA Transcripts) algorithm^19^, and we found that for most immune cell types, the relative quantity of immune cells was the highest in the H subtype and was the lowest in the L subtype (Fig. 1f). Downregulation of the major histocompatibility class I antigen (MHC-I) molecules results in immune evasion and resistance to ICI therapy^20^. Among the three melanoma subtypes, the MHC-I genes showed the highest expression levels in the H subtype and the lowest expression levels in the L subtype (Fig. 1g). Cytotoxic CD8+ T cells recognize tumor antigens presented on MHC-I molecules and have essential roles in anti-tumor immunity. Therefore, we examined the expression of the marker of cytotoxic CD8+ T cells and the activity of these cells^21^. We found that the H subtype had the highest expression levels of *CD8A* (encoding CD8), *IFNG* (encoding interferon-γ), and *TNF* (encoding tumor necrosis factor-α), whereas the L subtype had the lowest expression levels of these three genes (Fig. 1h). Moreover, the H subtype showed the highest levels, while the L subtype showed the lowest levels of *CD274* (encoding programmed cell death 1 ligand, PD-L1) and *CTLA4* (encoding cytotoxic T‐lymphocyte antigen 4) (Fig. 1i). These data suggest that the H subtype might respond better to anti-PD-L1 or anti-CTLA4 immunotherapy than the other two subtypes, considering that PD-L1 and CTLA4 expression levels tend to be positively associated with immunotherapeutic responsiveness^22^. Consistent with previous findings that elevated immune activity correlates with favorable clinical outcomes^23^, the H subtype had a significantly better overall survival (OS) than the M and L subtypes (log-rank *P* = 1.5 × 10^−4^, Fig. 1j).

Genetic mutations can generate tumor neoantigens that stimulate the immune response and enhance the response to ICIs. Previous studies have demonstrated that tumor mutational burden (TMB) is associated with neoantigen load^24,25^, and that patients with high TMB are more likely to benefit from ICIs and have a better survival rate^26–28^. However, the analysis of mutations of the SKCM genome in the TCGA database revealed no significant difference in TMB among the three immune subtypes (Supplementary Fig. 1a, b). These results were consistent with a recent study, which also showed no significant association between TMB and an immune-related signature in TCGA melanoma samples^14^.

GSEA of KEGG (Kyoto Encyclopedia of Genes and Genomes)^29^ pathways enabled us to identify pathways enriched in different immune subtypes of melanoma (Supplementary Fig. 2a). Consistent with elevated immune activity in the H subtype, we found that immune-related pathways, including the T cell receptor signaling pathway, the PD-L1 expression and PD-L1 checkpoint pathway, the TNF signaling pathway, and the Toll-like receptor signaling pathway, were highly active in the H subtype (Supplementary Fig. 2a). Moreover, two cancer-associated pathways, the NF-κB signaling pathway and the JAK-STAT signaling pathway, were also hyperactivated in the H subtype (Supplementary Fig. 2a), suggesting that the activities of these two tumor signaling pathways are associated with SKCM immunity. We also performed GSEA on gene ontology and found that the H subtype was enriched in immunoglobulin-mediated immune response, MHC protein complex, and T cell receptor complex (Supplementary Fig. 2b), which could further explain why the H subtype had high immunity and favorable clinical outcomes.

### Establishment of the TIR signature as a prognostic factor in melanoma

**Fig. 2:**
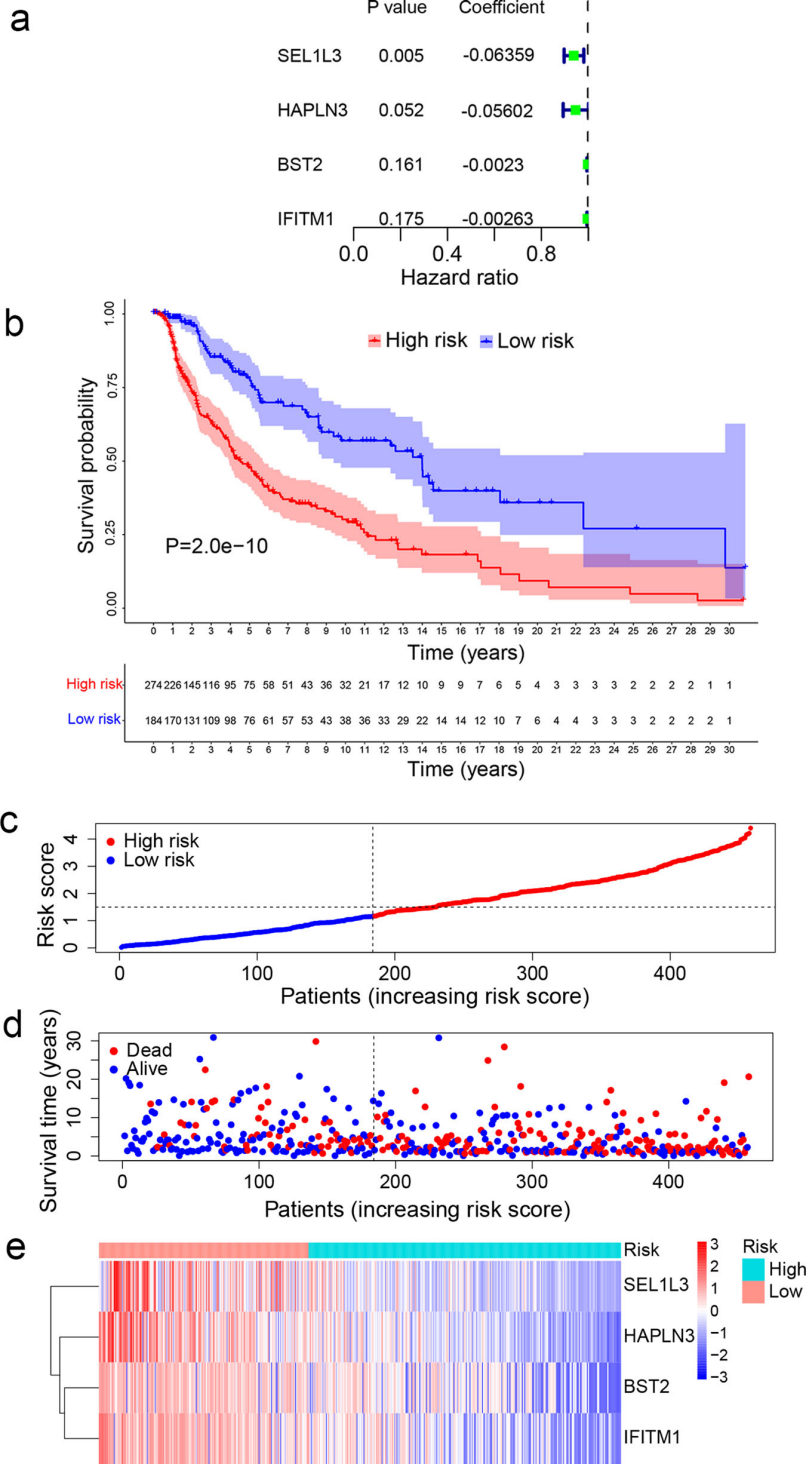
Establishment of a tumor immune-related prognostic signature (TIR signature) for melanoma. **a** Cox proportional hazard regression model was used to construct a prognostic signature consisting of four genes (*SEL1L3*, *HAPLN3*, *BST2*, and *IFITM1*). The coefficient (*β*), *P* value, and hazard ratio of each gene were calculated by the model. **b** Kaplan–Meier curves of the overall survival of patients with high TIR risk scores and those with low TIR risk scores. Statistical significance was determined by the log-rank test. **c** All TCGA SKCM patients were ranked from the lowest to the highest TIR risk score. **d** The overall survival time and survival status of individual patients. The black dotted line represents the median TIR risk score that divides patients into low-risk and high-risk groups. **e** Heatmap of *SEL1L3*, *HAPLN3*, *BST2*, and *IFITM1* expression levels. Rows represent genes and columns represent patients.

**Fig. 3:**
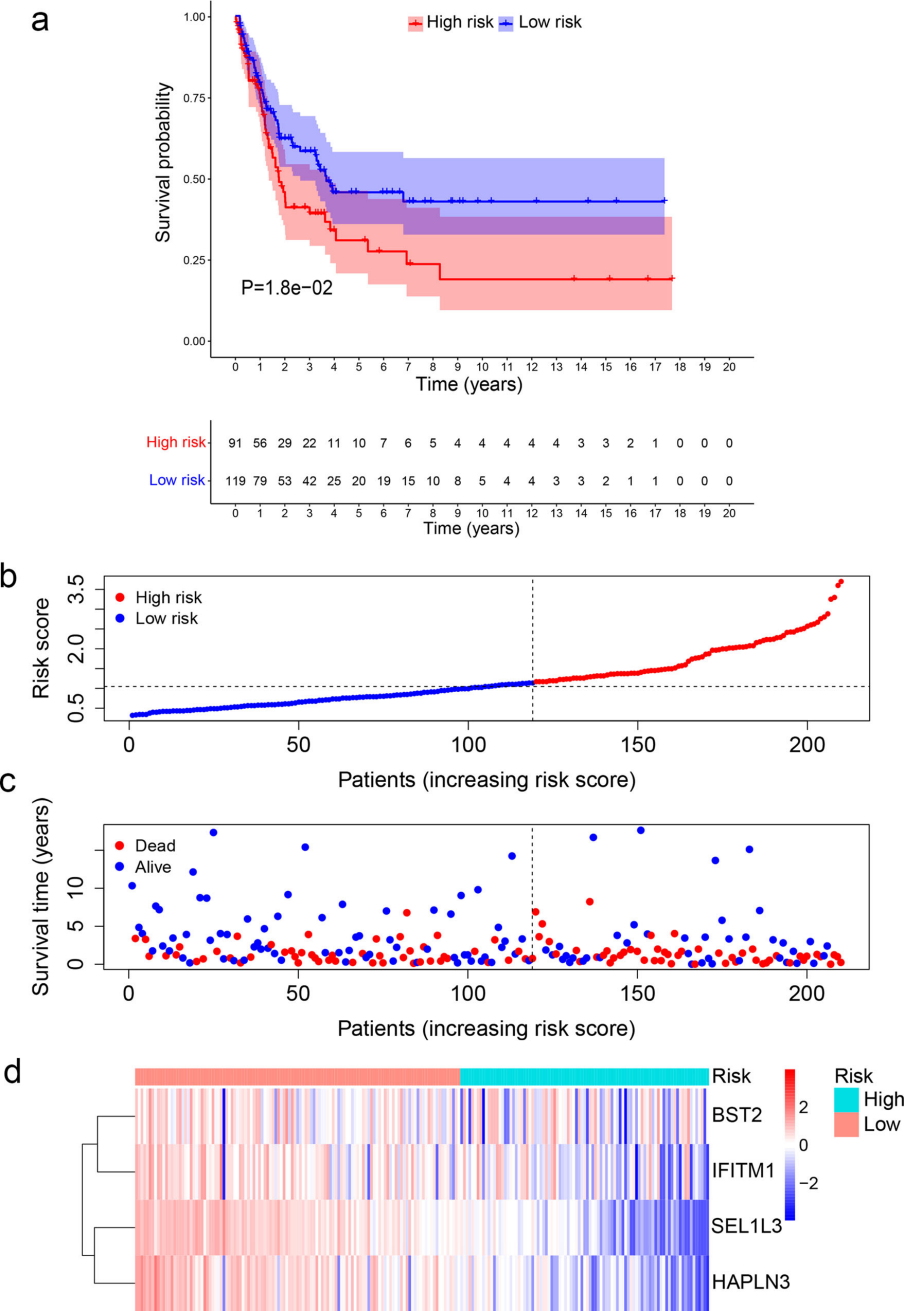
Validation of the TIR signature for survival prediction in an independent cohort. **a** Kaplan–Meier curves of the survival rate of patients in the GSE65904 cohort with high TIR risk scores and those with low TIR risk scores. Statistical significance was determined by the log-rank test. **b** All patients in the GSE65904 cohort were ranked from the lowest to the highest TIR risk score. **c** The overall survival time and survival status of individual patients in the GSE65904 cohort. The black dotted line represents the median TIR risk score that divides patients into low-risk and high-risk groups. **d** Heatmap of *SEL1L3*, *HAPLN3*, *BST2*, and *IFITM1* expression levels. Rows represent genes and columns represent patients in the GSE65904 cohort.

To facilitate the implementation of the above subtypes in clinical practice, we sought to build a simple gene signature that captures these immune subtypes. By comparing the mRNA levels between the high-immunity subtype and the low-immunity subtype, we identified a total of 450 differentially expressed genes (DEGs) associated with immune infiltration and immune activity (Supplementary Data 2). To explore the relationships between these DEGs and the survival outcome of SKCM patients, we performed the univariate Cox regression analysis by using the survival R package. To avoid overfitting the prognostic model, we used the Least Absolute Shrinkage and Selection Operator (LASSO) Cox regression model (Supplementary Fig. 3a, b). By performing the stepwise multiple Cox regression analysis, we identified a TIR signature consisting of four genes, *SEL1L3* (encoding SEL1L family member 3), *HAPLN3* (encoding hyaluronan and proteoglycan link protein 3), *BST2* (encoding bone marrow stromal cell antigen 2), and *IFITM1* (encoding interferon-induced transmembrane protein 1) (Fig. 2a). The TIR risk score of each SKCM patient was calculated based on the expression levels and the regression coefficients of the four genes (see “Methods” section). By using the median risk score as the threshold value, we divided the SKCM patients in the TCGA cohort into the high-risk group and the low-risk group. Kaplan–Meier analysis showed that patients with high TIR risk scores had worse OS than those with low TIR risk scores (log-rank *P* = 2 × 10^−10^, Fig. 2b). We then generated the risk curve and scatterplot for the TIR risk score and the survival status of individual SKCM patients in the TCGA cohort, finding that the risk coefficient and mortality of patients with high TIR risk scores were higher than those with low TIR risk scores (Fig. 2c, d). The heatmap of the TIR signature in SKCM patients showed that *SEL1L3*, *HAPLN3*, *BST2*, and *IFITM1* were highly expressed in the low-risk group (Fig. 2e). To further validate the prognostic value of the TIR signature obtained from the TCGA melanoma cohort, we tested this signature in an independent melanoma dataset (GSE65904)^30^ (Supplementary Data 3), finding that patients with high TIR risk scores had worse OS, higher risk coefficient and mortality, and lower expression levels of *SEL1L3*, *HAPLN3*, *BST2*, and *IFITM1* than those with low TIR risk scores (Fig. 3a–d).

**Fig. 4:**
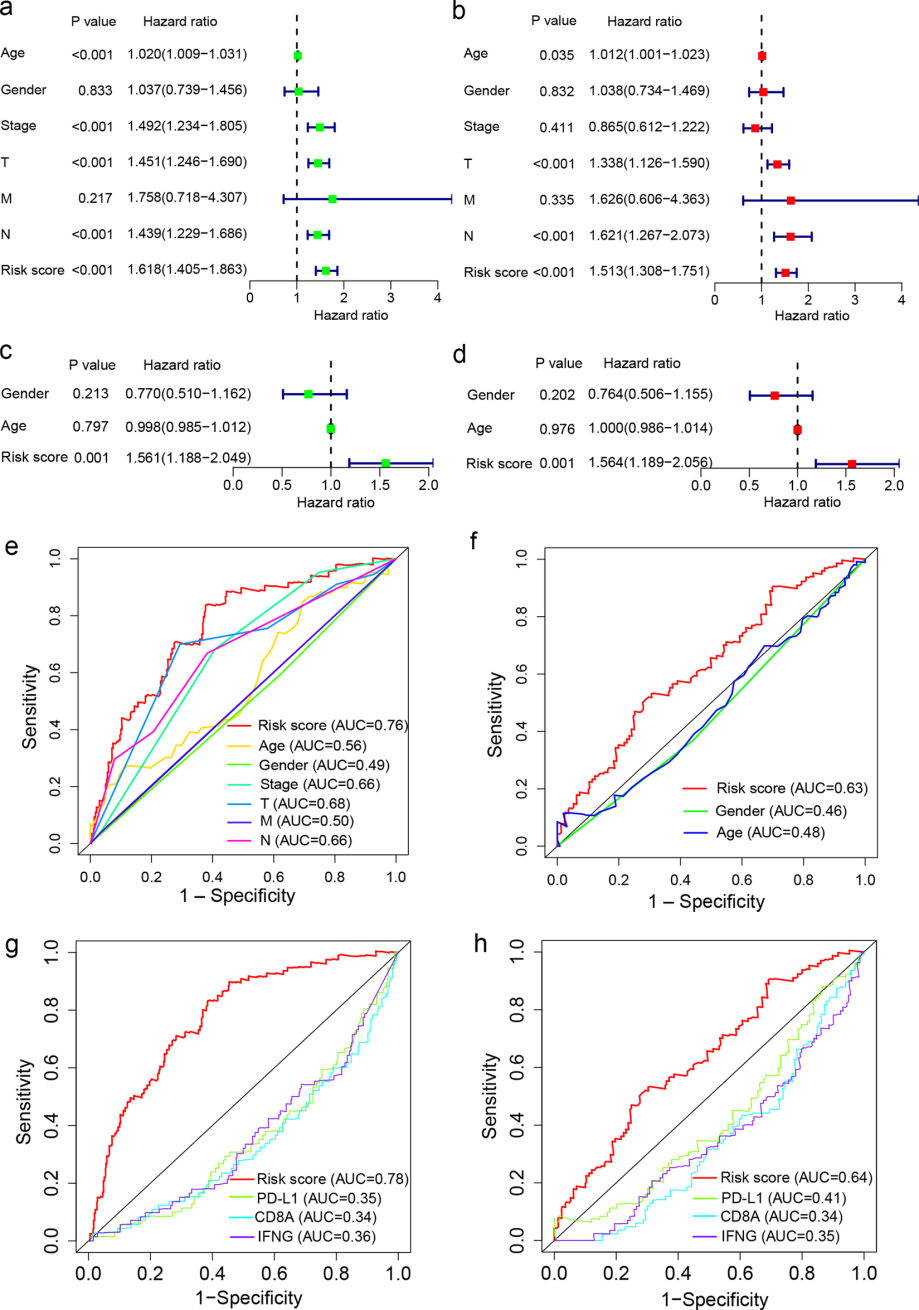
The Cox regression and ROC analyses of the TIR signature in predicting the survival of melanoma patients. **a**, **b** The univariate (**a**) and multivariate (**b**) Cox regression analyses of the TIR risk score, age, gender, stage, and TNM classification in the TCGA cohort. **c**, **d** The univariate (**c**) and multivariate (**d**) Cox regression analysis of the TIR risk score, age, and gender in the GEO validation set GSE65904. In **a**–**d**, the colored blocks represent the hazard ratio and the horizontal bars extend from the lower limit to the upper limit of the 95% confidence interval of the estimate of the hazard ratio. **e** Receiver operating characteristics (ROC) curves of the sensitivity and specificity of the TIR signature in predicting the overall survival (OS) of patients in the TCGA cohort. The area under the curve (AUC) values for the TIR risk score, age, gender, stage, and TNM classification were calculated based on the ROC curves of the TCGA cohort. **f** The AUC values for the TIR risk score, age, and gender were calculated based on the ROC curves of the GEO validation cohort GSE65904. **g** ROC curves of the sensitivity and specificity of the TIR signature and other biomarkers in predicting the OS of patients in the TCGA cohort. **h** ROC curves of the sensitivity and specificity of the TIR signature and other biomarkers in predicting the OS of patients in the GEO validation cohort GSE65904.

Next, we performed univariate and multivariate Cox regression analyses to assess whether the TIR risk score was prognostic independently of clinicopathological factors, including the age, gender, stage, and TNM classification. The hazard ratio (HR) of the TIR risk score and the 95% confidence interval (CI) were 1.618 and 1.405–1.863 in the univariate Cox regression analysis (*P* < 0.001), and were 1.513 and 1.308–1.751 in the multivariate Cox regression analysis (*P* < 0.001), respectively, suggesting that the TIR risk score was an independent prognostic factor in melanoma patients in the TCGA cohort (Fig. 4a, b). Further, by performing univariate and multivariate Cox regression analyses of the GSE65904 dataset, we validated the TIR risk score as an independent factor prognostic of survival (Fig. 4c, d).

To evaluate the sensitivity and specificity of the TIR risk score, we performed the time-dependent receiver operating characteristics (ROC) analysis of the TCGA cohort. The area under the ROC curve (AUC) of the TIR risk score was 0.76, which was higher than those of clinicopathological factors (Fig. 4e). Similar results were obtained from the GSE65904 cohort (Fig. 4f). We also performed ROC analyses of the TCGA cohort and the GSE65904 cohort to compare the TIR risk score with other biomarkers, including PD-L1, CD8A, and IFNG^31–33^, finding that the TIR signature had higher AUC than other biomarkers in both cohorts (Fig. 4g, h). These results suggest that the TIR signature can better predict the survival of melanoma patients.

### Correlation of the TIR signature with the immune infiltration, anti-CTLA4 immunotherapy response, and gene methylation in melanoma

Given that the TIR signature was established based on tumor immunity, we analyzed the correlation between the four genes in the TIR signature and the infiltration of immune cell types in melanoma by using the Tumor IMmune Estimation Resource (TIMER; https://cistrome.shinyapps.io/timer/) algorithm^34^. As shown in Supplementary Fig. 4, the expression levels of *SEL1L3*, *HAPLN3*, *BST2*, and *IFITM1* were positively associated with the infiltration levels of CD8+ T cells, CD4+ T cells, macrophages, and dendritic cells. We also found significant correlations of these four genes with the mRNA levels of CTLA4, PD-L1 (Supplementary Fig. 5a), and MHC-I molecules, including HLA-A, HLA-B, and HLA-C (Supplementary Fig. 5b).

**Fig. 5:**
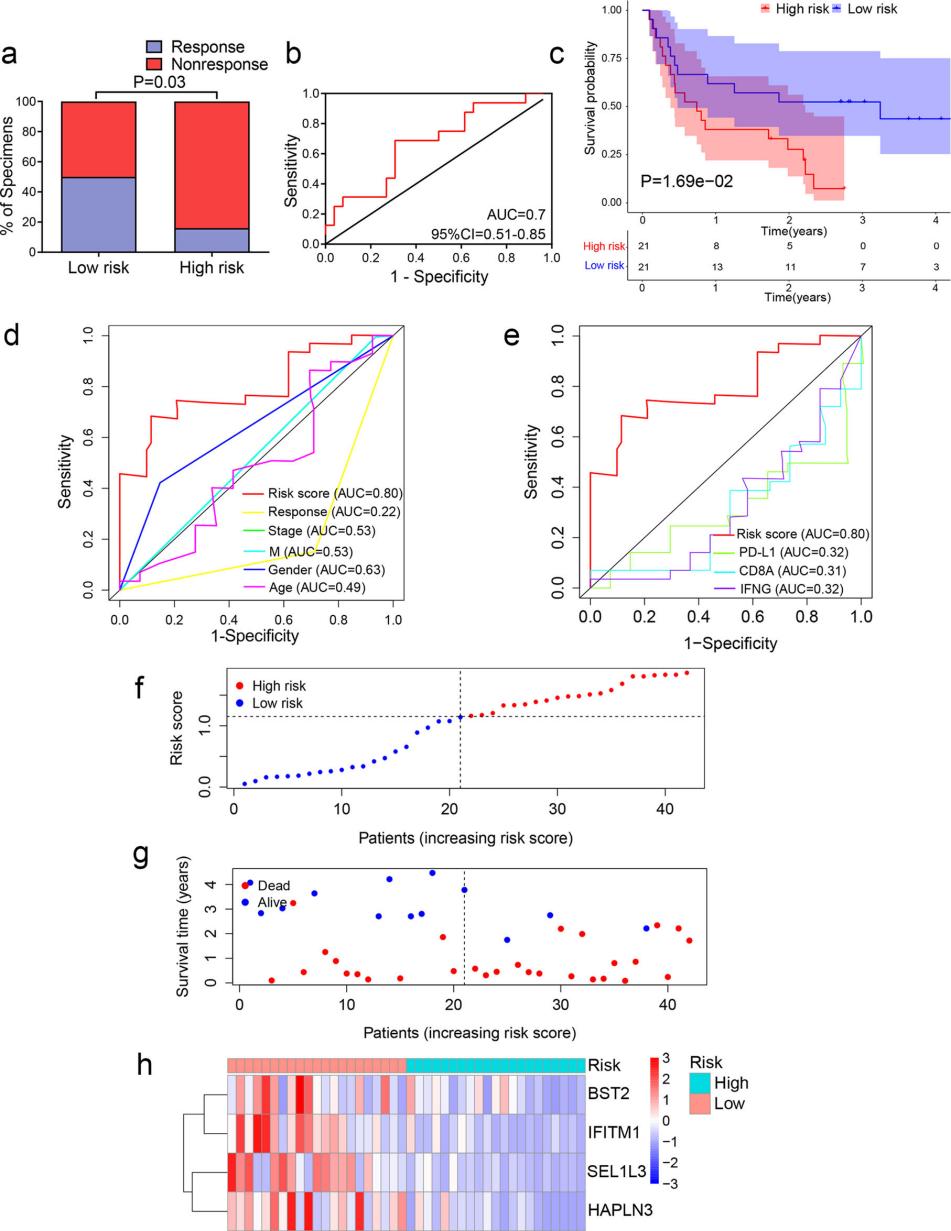
The TIR signature can predict the response of melanoma patients to anti-CTLA4 therapy. **a** Anti-CTLA4 responses (response or nonresponse) of melanoma patients with high or low TIR risk scores in the dbGap cohort (*n* = 42). Statistical significance was determined by the chi-square test. **b** ROC curves evaluating the accuracy of the TIR signature for predicting the anti-CTLA4 response of patients in the dbGap cohort. **c** Kaplan–Meier curves of the overall survival of patients in the dbGap cohort with high TIR risk scores and those with low TIR risk scores. Statistical significance was determined by the log-rank test. **d** ROC curves of the TIR signature and clinicopathological factors in predicting the OS of patients in the dbGap cohort. **e** ROC curves of the sensitivity and specificity of the TIR signature and other biomarkers in predicting the OS of patients in the dbGap cohort. **f** All patients in the dbGap cohort were ranked from the lowest to the highest TIR risk score. **g** The overall survival time and survival status of individual patients in the dbGap cohort. The black dotted line represents the median TIR risk score that divides patients into low-risk and high-risk groups. **h** Heatmap of *SEL1L3*, *HAPLN3*, *BST2*, and *IFITM1* expression levels. Rows represent genes and columns represent patients in the dbGap cohort.

Next, we evaluated whether the TIR signature can predict the response to ICIs. By analyzing a melanoma cohort (database of Genotypes and Phenotypes (dbGaP) accession number: phs000452.v2.p1) with RNA-seq data and anti-CTLA4 (ipilimumab) therapy response information available from 42 patients^28^, we found that patients with low TIR risk scores had a higher anti-CTLA4 response rate than patients with high TIR scores (chi-square test, *P* = 0.03, Fig. 5a). The ROC curve also showed that the TIR signature could predict the ipilimumab therapy response of melanoma patients (AUC = 0.7, 95% CI = 0.51–0.85, Fig. 5b). By performing the Kaplan–Meier analysis of this cohort, we found that patients with high TIR risk scores had a worse survival rate than patients with low TIR risk scores (log-rank *P* = 0.0169, Fig. 5c). ROC curves for survival prediction showed that the TIR risk score had higher AUC than clinicopathological factors (Fig. 5d) and other biomarkers (PD-L1, CD8A, and IFNG, Fig. 5e). The risk curve and scatterplot of this cohort demonstrated that the risk coefficient and mortality of patients with high TIR risk scores were higher than those with low TIR risk scores (Fig. 5f, g). The heatmap of the TIR signature in this cohort showed that the expression levels of *SEL1L3*, *HAPLN3*, *BST2*, and *IFITM1* were higher in the low-risk group than in the high-risk group (Fig. 5h). Collectively, these results suggest that the four-gene TIR signature is a predictor of anti-CTLA4 immunotherapy response and survival of melanoma patients.

**Fig. 6:**
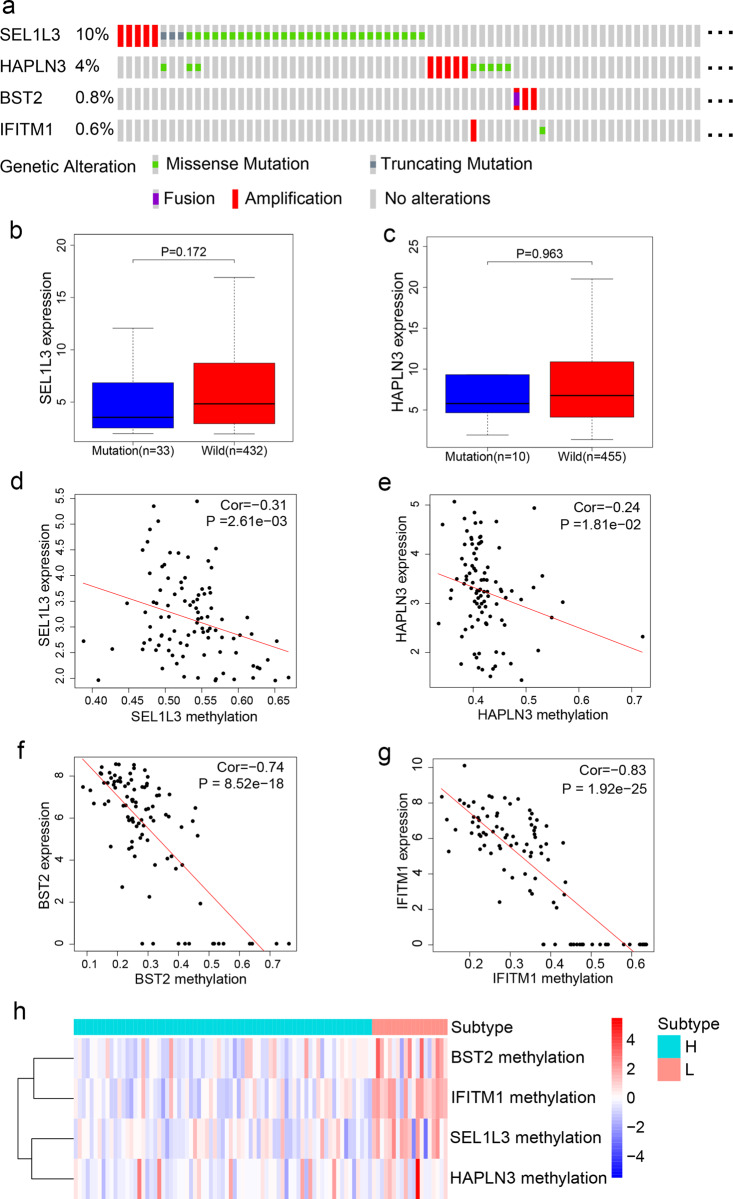
Multi-omics analyses of the four genes in the TIR signature. **a** The mutational status of *SEL1L3*, *HAPLN3*, *BST2*, and *IFITM1* in TCGA SKCM patients. Data were from the cBioPortal database. **b**, **c** mRNA levels of *SEL1L3* (**b**) and *HAPLN3* (**c**) in the TCGA SKCM patients stratified by the mutation status. Statistical significance was determined by the Wilcoxon test. *SEL1L3* and *HAPLN3* were not analyzed due to the very small sample size in the mutation group. The boxplot consists of a box and a set of whiskers. The box is drawn from the first quartile (25th percentile) to the third quartile (75th percentile) with a horizontal line drawn in the middle to denote the median. The lowest point is the minimum of the dataset and the highest point is the maximum of the dataset. **d**–**g** Correlations between mRNAs levels and methylation levels of *SEL1L3* (**d**), *HAPLN3* (**e**), *BST2* (**f**), *IFITM1* (**g**) in TCGA SKCM patients. Statistical significance was determined by the Spearman correlation test. **h** Heatmap of *SEL1L3*, *HAPLN3*, *BST2*, and *IFITM1* methylation levels in H and L subtypes. Rows represent genes and columns represent patients.

By analyzing the TCGA genomics data of *SEL1L3*, *HAPLN3*, *BST2*, and *IFITM1*, we found that the gene alteration rates in melanoma were 10% for *SEL1L3*, 4% for *HAPLN3*, 0.8% for *BST2*, and 0.6% for *IFITM1* (Fig. 6a), and these alterations were not recurrent. There was no significant difference in *SEL1L3* and *HAPLN3* gene expression levels between the mutated group and the wild-type group (Fig. 6b, c). Instead, the DNA methylation levels of *SEL1L3*, *HAPLN3*, *BST2*, and *IFITM1* genes showed significant inverse correlations with their mRNA levels (Fig. 6d–g). Moreover, the methylation levels of *BST2* and *IFITM1* were higher in the L subtype than in the H subtype (Fig. 6h), suggesting that DNA hypermethylation may underlie the underexpression of *BST* and *IFITM1* in the L subtype (low immunity, high risk). Consistently, patients with *IFITM1* or *BST2* hypermethylation, but not *SEL1L3* or *HAPLN3* hypermethylation, had significantly worse OS than the hypomethylation groups (Supplementary Fig. 6a–d).

## Discussion

**Fig. 7:**
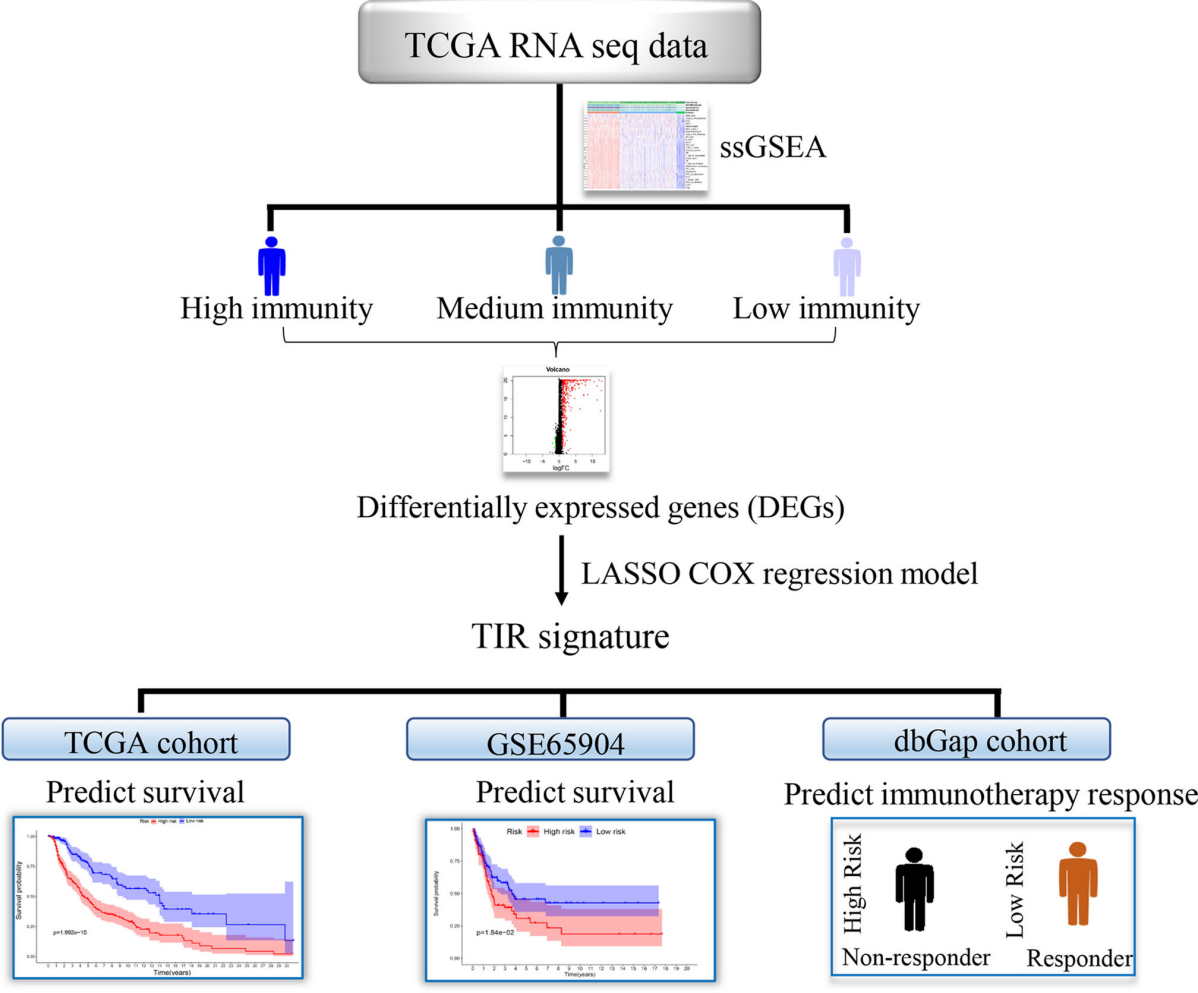
Graphical summary. The flow chart illustrates the process followed to identify a four-gene signature that predicts survival and anti-CTLA4 immunotherapeutic responses based on immune classification of melanoma.

Melanoma is highly heterogeneous in clinical, dermatological, and histopathological aspects^35^. Several clinical parameters, such as gender, age, stage, and Breslow thickness, influence the prognosis of patients with melanoma. Previous studies have suggested the prognostic value of immune cell infiltration in melanoma^36,37^. In this study, we identified a four-gene signature associated with survival and anti-CTLA4 immunotherapeutic responses based on the immune subtype classification of melanoma (Fig. 7). First, we used the ssGSEA to quantitate the enrichment level of 28 immune-associated gene sets in each melanoma sample in the TCGA database. Then, based on the enrichment score of each sample, we classified the patients into three immune subtypes (high-, medium-, and low-immunity). Next, we identified subtype-specific features, including immune and stromal cell infiltration levels, pathways, and gene ontology. We further identified DEGs by comparing the transcriptomic data between melanoma patients with different immune subtypes. A four-gene signature, named the TIR signature, was established using the LASSO Cox regression model, and the TIR signature was found to be highly associated with the expression of MHC-I, the activity of CD8+ T cells, and immune infiltration in melanoma patients.

Cancer immunotherapies by ICIs, such as antibodies against PD-L1, PD-1, and CTLA4, have achieved notable success by blocking immune-inhibitory signals and enabling patients to produce antitumor immunity^38^. However, less than one-third of patients respond to ICI treatment, and the identification of predictive biomarkers and regulators of immunotherapy responses is highly needed^39^. When applied to a cohort of melanoma patients treated with ipilimumab^28^, the TIR signature could predict the therapy response and the survival outcome better than known biomarkers, suggesting the potential use of this four-gene signature to guide the treatment of melanoma patients with ipilimumab. We also analyzed a cohort of melanoma patients treated with the PD-1 antibody (pembrolizumab or nivolumab)^40^, finding no significant association of the TIR signature with the therapy response. Considering the relatively small number of samples in this cohort (*n* = 28), the analysis of larger cohorts of patients with anti-PD-1 (or anti-PD-L1) therapy response information available is of future interest.

Although there were some genetic alterations of the four genes in the TIR signature, no significant difference in gene expression was found between the wild-type group and the mutation group. Instead, the methylation levels of the four genes showed a significant negative correlation with the mRNA levels of *SEL1L3*, *HAPLN3*, *BST2*, and *IFITM1*, with particularly high correlation coefficients for *BST2* and *IFITM1*. IFITM1 is a member of the IFN-induced transmembrane protein family. Interestingly, a recent study revealed an association between aberrant methylation of *IFITM1* and poor disease-specific survival in patients with acral melanoma, an aggressive type of cutaneous melanoma^41^. BST2 (also known as CD317), a cell surface glycoprotein, was previously reported to be induced by type I interferon in response to viral infection^42,43^. To date, SEL1L3 and HAPLN3 have not been implicated in innate or adaptive immunity. Future studies are warranted to uncover the function and mechanism of action of IFITM1, BST2, SEL1L3, and HAPLN3 in antitumor immunity and immunotherapy response.

In summary, we classified melanoma patients into three subtypes based on immune activity and identified a four-gene TIR signature that significantly correlates with the survival of melanoma patients. The TIR signature is an independent prognostic factor and predicts survival better than other factors. Further, the TIR signature can predict the response of melanoma patients to anti-CTLA4 therapy and their survival outcome, with higher accuracy than other biomarkers. Thus, the TIR signature holds promise as a clinical biomarker. Compared with complex gene signatures, it is much easier to implement these four genes in clinical practice. Whether and how the genes in the TIR signature functionally regulate antitumor immunity warrants future investigation.

## Methods

### Collection of SKCM data and clustering

The fragments per kilobase of transcript per million mapped reads (FPKM) values of the SKCM RNA-sequencing data and corresponding clinical data were downloaded from TCGA (https://portal.gdc.cancer.gov), and the sample size is 471. Based on 28 immune-associated gene sets^17^ representing immune cell subsets, immune-related functions, and immune-related pathways, we used the ssGSEA method of the R software Gene Set Variation Analysis (GSVA) package to calculate the ssGSEA score of each SKCM sample^16^. According to the ssGSEA scores of the 28 immune-associated gene sets, we performed hierarchical clustering of SKCM samples by using the “hclust” function (R package) and classified them into low-, medium-, and high-immunity subtypes by using the “cutree” function (R package). The accession number of the dataset from Gene Expression Omnibus (GEO) is GSE65904, and the sample size is 214. The accession number of the dataset from dbGap is phs000452.v2.p1, and the sample size is 42.

### Evaluation of tumor purity, immune cell infiltration level, and stromal content in SKCM

To verify the ssGSEA analysis and to draw the clustering heat map, we used ESTIMATE^18^ to calculate the tumor purity, immune cell infiltration level (immune score), and stromal content (stromal score) of each SKCM sample based on the RNA-sequencing data.

### Comparison of the proportions of immune cells between SKCM subtypes

CIBERSORT^19^ was used to calculate the proportions of 15 human immune cell subsets. We set 1000 permutations and *P* < 0.05 as the criteria for the successful deconvolution of an SKCM sample. We compared the proportions of the 15 immune cells between SKCM subtypes by using the Mann–Whitney *U* test.

### Survival analyses

We performed survival analyses of the TCGA, GSE65904, and dbGap cohorts. Kaplan–Meier curves were plotted to assess the differences in OS. The log-rank test was used to evaluate the significance of OS differences with a threshold of *P* < 0.05.

### Gene-set enrichment analysis

We performed the GSEA of the TCGA SKCM data by GSEA (R implementation)^44,45^. This analysis identified the KEGG^29^ pathways and gene ontology that were enriched in the high-immunity subtype or the low-immunity subtype (false discovery rate, FDR < 0.05), respectively.

### Identification of DEGs

To identify DEGs between the high-immunity subtype and the low-immunity subtype, we used the limma R package to generate the FDR and the fold change (FC) for each gene. Genes with FDR <0.05 and |log_2_FC| ≥1 were defined as DEGs^46^.

### Establishment of a TIR signature for SKCM prognosis

Based on the clinical data of SKCM in TCGA, we performed the univariate Cox regression analysis by using the survival R package to screen DEGs for prognostic genes. For the construction of the TIR gene signature, DEGs with a *P* < 0.05 were selected, and the LASSO Cox regression model was used to prevent overfitting by using the glmnet R package. Then, the multivariate Cox proportional hazards regression model was used to generate the TIR signature consisting of four key prognostic genes, *SEL1L3*, *HAPLN3*, *BST2*, and *IFITM1*. The Cox model is expressed by the hazard function denoted by *h*(*t*, *X*). The hazard function can be interpreted as the risk of dying at time *t*, and it can be estimated as follows: *h*(*t*, *X*) = *h*_0_(*t*) × exp(*β*_1_*X*_1_ + *β*_2_*X*_2_ + … + *β*_*m*_*X*_*m*_), where *t* represents the survival time, *h*(*t*, *X*) is the hazard function determined by a set of covariates (*X*_1_, *X*_2_, … , *X*_*m*_), and the coefficients (*β*_1_, *β*_2_, … , *β*_*m*_) measure the impact of covariates. *h*_0_(*t*) is the baseline hazard rate of *h*(*t*, *X*) when all the *X* is zero. *h*(*t*, *X*) can be calculated by the “predict” function of R software. Thus, the TIR risk score of each melanoma patient was calculated using the “predict” function of R software based on the expression levels (*X*) and the regression coefficients (*β*) of the four genes. Next, the TIR signature was applied to the GSE65904 cohort and the dbGap cohort (accession number: phs000452.v2.p1) to validate the prognostic value of the TIR signature.

### Correlation analysis of the TIR signature with immune cell infiltration

The correlations of the four genes in the TIR signature with the infiltration levels of CD8+ T cells, CD4+ T cells, dendritic cells, and macrophages were analyzed using the TIMER algorithm (https://cistrome.shinyapps.io/timer/).

### Statistics and reproducibility

Statistical analyses were performed using R version 3.6.1. Differences between two groups of samples were compared using unpaired Student’s *t*-test (normally distributed) or the Mann–Whitney *U* test (non-normally distributed). Differences among three or more groups were compared using the Kruskal–Wallis test (non-normally distributed) or one-way analysis of variance (normally distributed). Spearman correlation analysis was used to calculate the correlation coefficient between two factors. Survival differences between different groups were compared using the Kaplan–Meier analysis and the log-rank test. Differences in response rates (i.e., ratios of responders to total patients) between different groups were compared using the chi-square test or Fisher’s exact test. To evaluate the specificity and sensitivity of the TIR signature, we generated the ROC curve by using the survivalROC package to calculate the AUC and 95% CI. For each ROC analysis, we included all patients with information available for survival and the factors (clinicopathological factors or other biomarkers) to be compared with the TIR risk score.

### Reporting summary

Further information on research design is available in the Nature Research Reporting Summary linked to this article.

## Supplementary information

Supplementary Information

Description of Additional Supplementary Files

Supplementary Data 1

Supplementary Data 2

Supplementary Data 3

Reporting Summary

## Data Availability

The datasets are available in the following public databases: TCGA (https://portal.gdc.cancer.gov), melanoma dataset; Gene Expression Omnibus (https://www.ncbi.nlm.nih.gov/gds/), accession number: GSE65904; and dbGap (https://www.ncbi.nlm.nih.gov/gap/), accession number: phs000452.v2.p1. The data behind the figures are available in the Supplementary Data.

## Code availability

R code used for data processing and analysis was submitted to GitHub repository (https://github.com/262062/TIR) and deposited to Zenodo (10.5281/zenodo.4536570).
