# Supplementary material for: A four-gene signature predicts survival and anti-CTLA4 immunotherapeutic responses based on immune classification of melanoma
Source: Commun Biol. 2021 Mar 22;4:383. doi: 10.1038/s42003-021-01911-x (PMC7985195; doi:10.1038/s42003-021-01911-x)
Supplement: Supplementary file 1 — Supplementary Information [file 42003_2021_1911_MOESM1_ESM.pdf]

A four-gene signature predicts survival and anti-CTLA4 immunotherapeutic responses based on immune classification of melanoma

Ying Mei[1], Mei-Ju May Chen[2], Han Liang[2,3,4], Li Ma[1,4,*]

[1] Department of Experimental Radiation Oncology, The University of Texas MD Anderson Cancer Center, Houston, Texas 77030, USA

[2] Department of Bioinformatics and Computational Biology, The University of Texas MD Anderson Cancer Center, Houston, Texas 77030, USA

[3] Department of Systems Biology, The University of Texas MD Anderson Cancer Center, Houston, Texas 77030, USA

[4] The University of Texas MD Anderson Cancer Center UTHealth Graduate School of Biomedical Sciences, Houston, Texas 77030, USA

* Correspondence: lma4@mdanderson.org

Figure S1

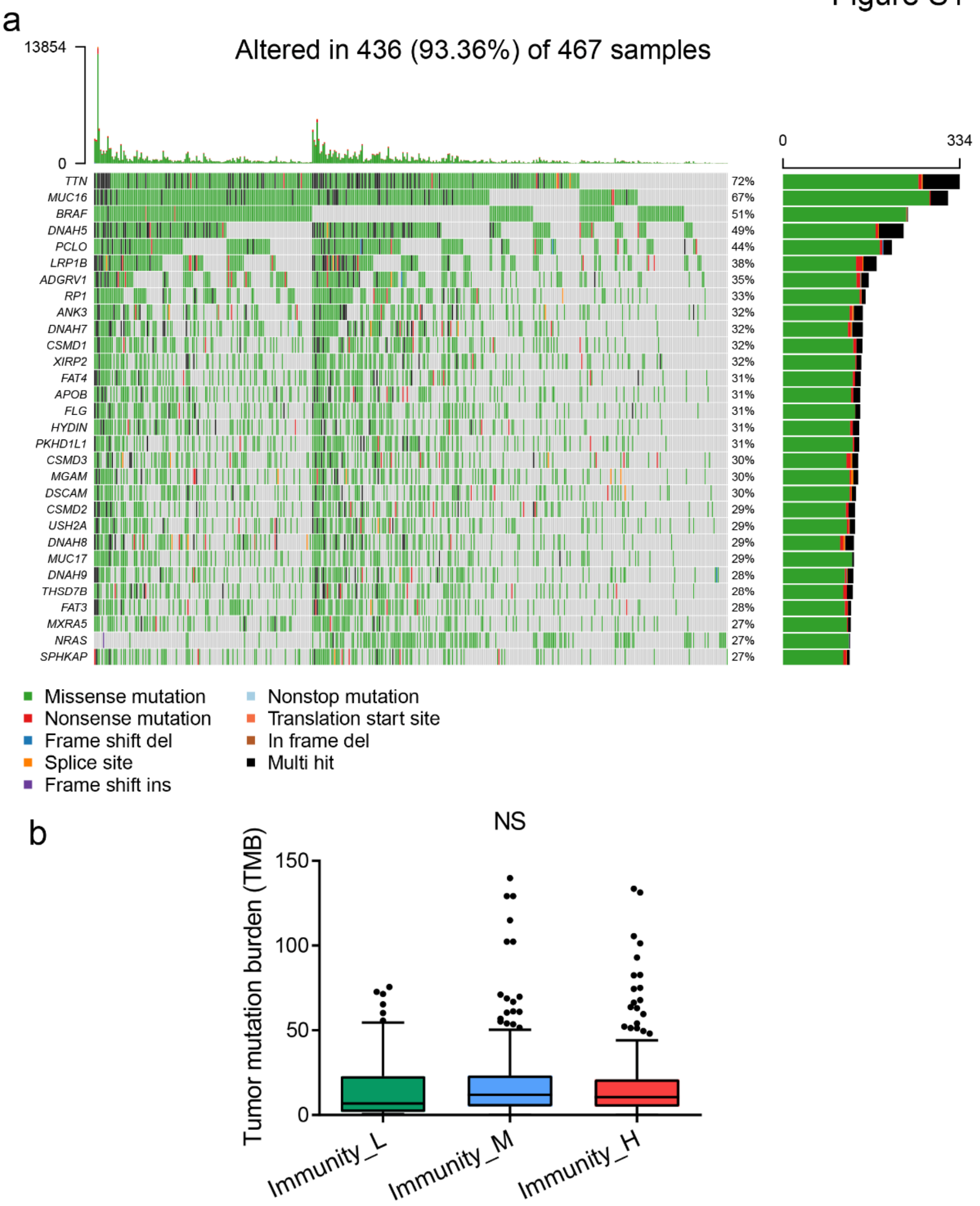

Supplementary Fig. 1. Mutational analysis of TCGA SKCM patients.

(a) The mutation status of the top 30 mutated genes in TCGA SKCM patients. The mutation frequencies of individual patients are shown next to the heatmap.

(b) Comparison of tumor mutation burden (TMB) in three immune subtypes of melanoma. Statistical significance was determined by the Kruskal-Wallis test. NS: not significant.

Figure S2

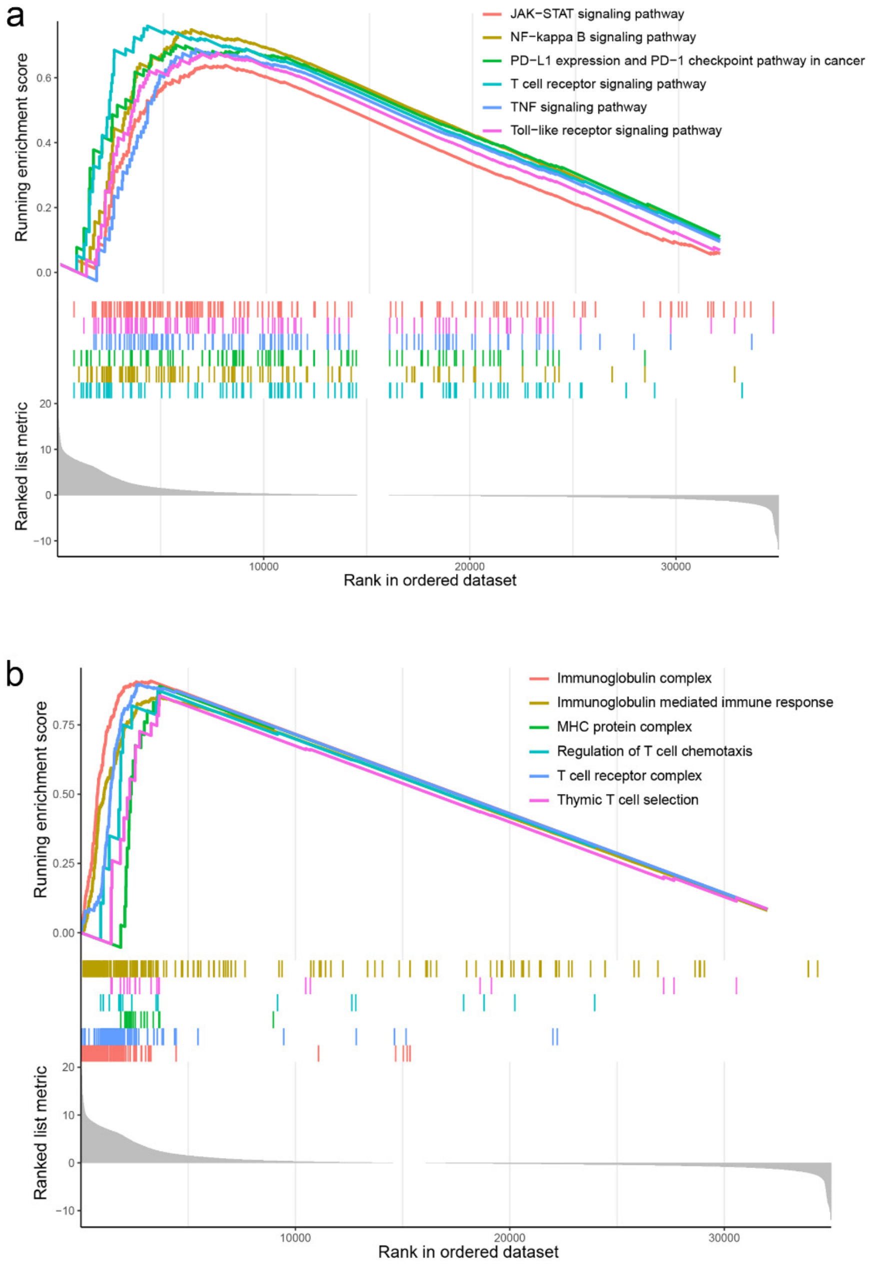

Supplementary Fig. 2. Identification of melanoma immune subtype-specific pathways and gene ontology.

(a) Gene-set enrichment analysis (GSEA) of the SKCM samples identified the KEGG pathways that were enriched in H and L subtypes, respectively.

(b) GESA identified the gene ontology that was enriched in H and L subtypes, respectively.

Figure S3

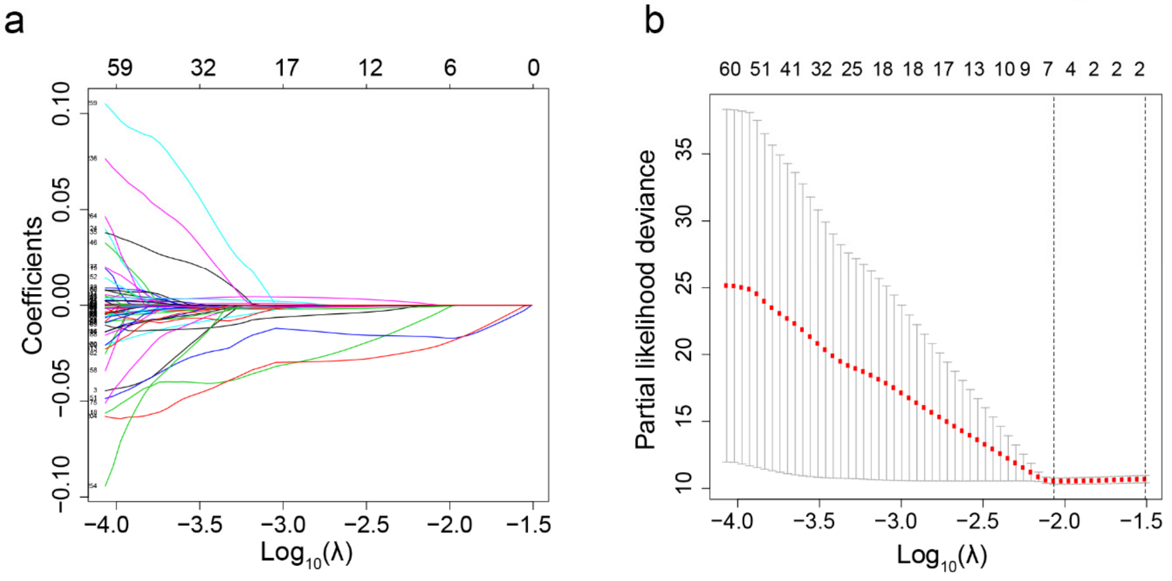

Supplementary Fig. 3. LASSO Cox analysis of differentially expressed genes.

(a) Least absolute shrinkage and selection operator (LASSO) coefficient profiles of 60 key genes identified by the model.

(b) The partial likelihood deviance was plotted against log (λ), where λ is the tuning parameter. The LASSO Cox analysis identified six differentially expressed genes that were associated with survival most significantly.

Figure S4

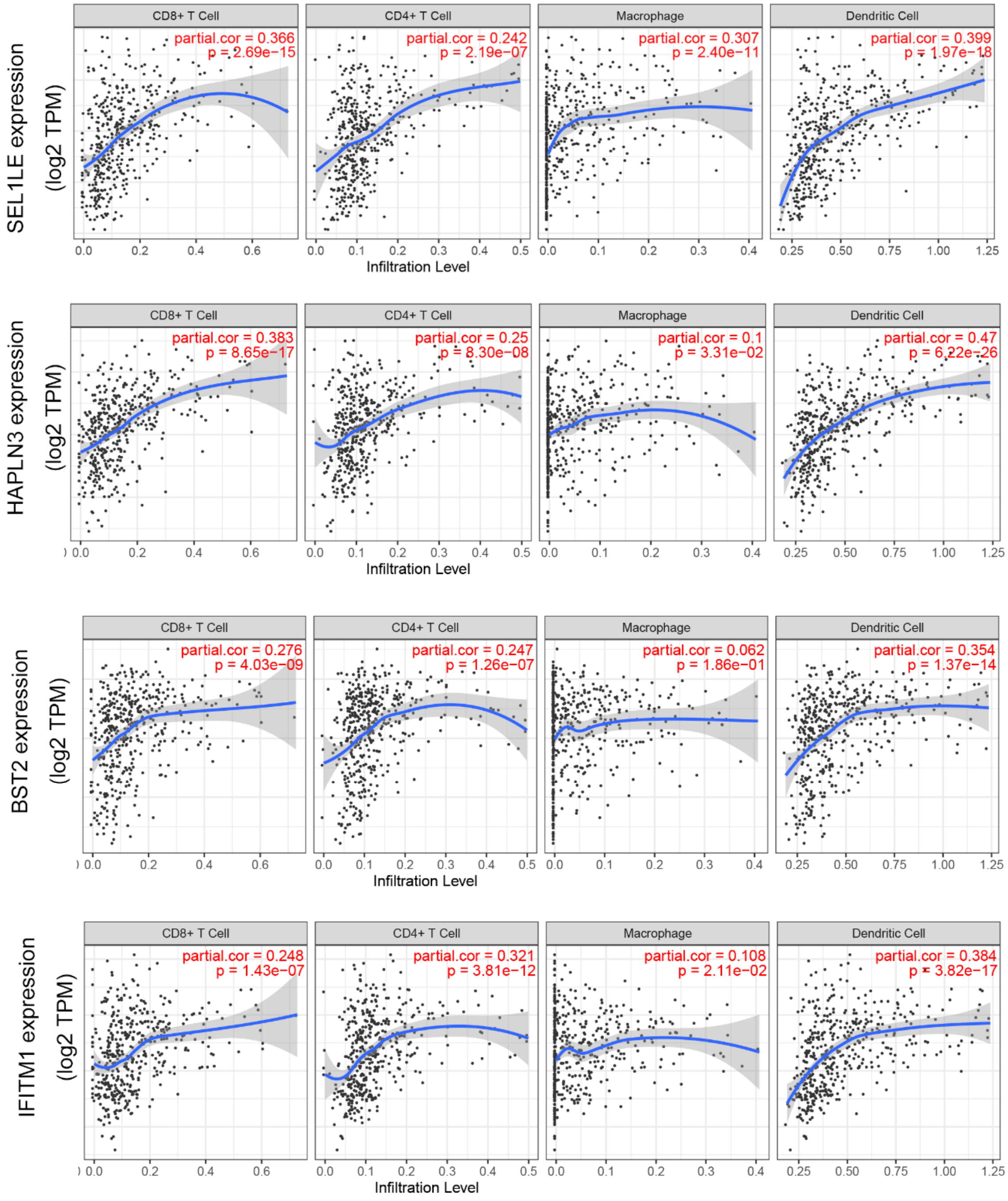

Supplementary Fig. 4. Correlations of *SEL1L3*, *HAPLN3*, *BST*2, and *IFITM1* mRNA levels with the infiltration levels of immune cells (CD8+ T cells, CD4+ T cells, macrophages, and dendritic cells).
Statistical significance was determined by the Spearman correlation test.

Figure S5

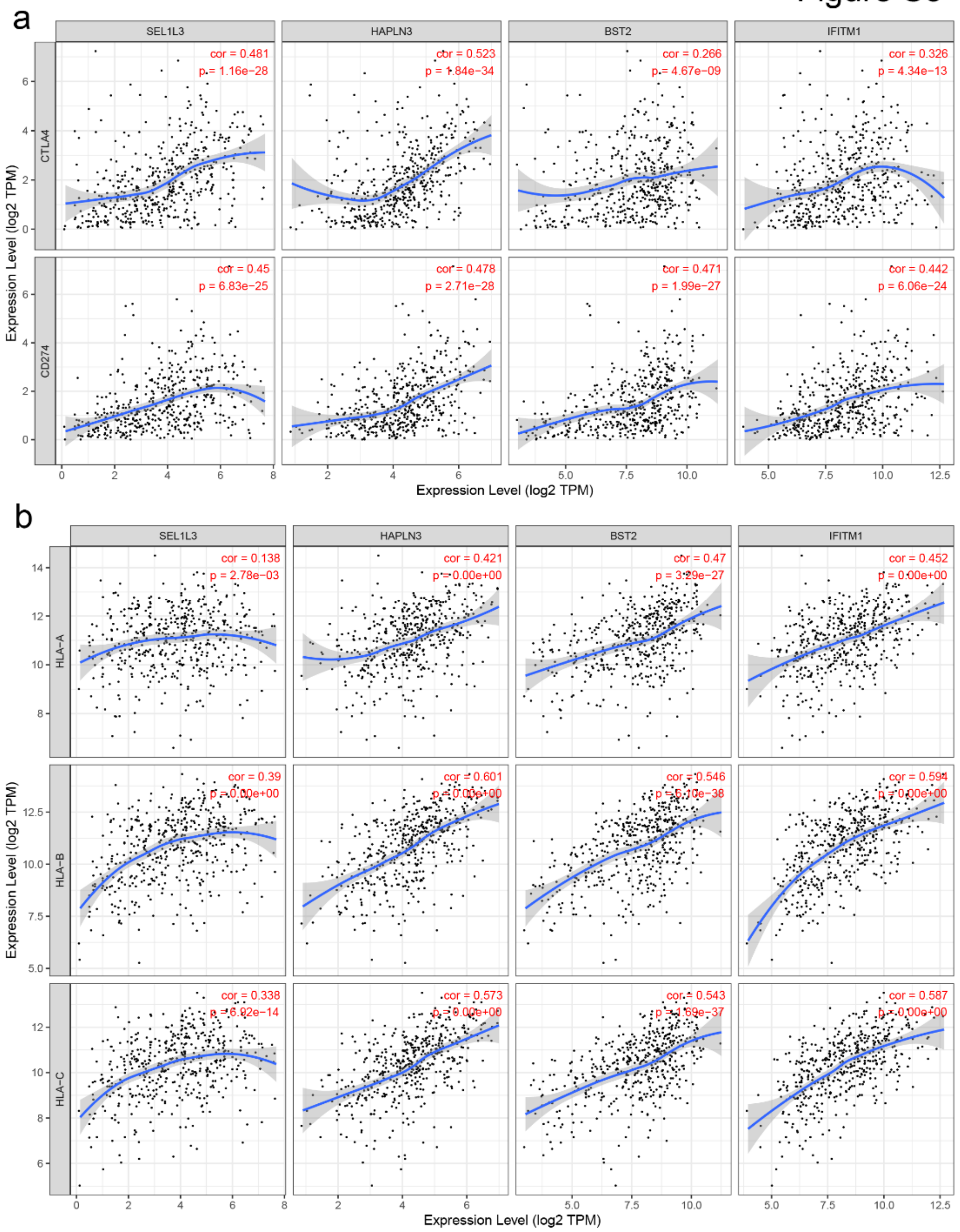

Supplementary Fig. 5. Correlations between the TIR gene signature and immune biomarkers.

(a) Correlations of *SEL1L3*, *HAPLN3*, *BST2*, and *IFITM1* mRNA levels with *CD274* and *CTLA4* mRNA levels. Statistical significance was determined by the Spearman correlation test.

(b) Correlations of *SEL1L3*, *HAPLN3*, *BST2*, and *IFITM1* mRNA levels with mRNA levels of MHC-I molecules including HLA-A, HLA-B, and HLA-C. Statistical significance was determined by the Spearman correlation test.

Figure S6

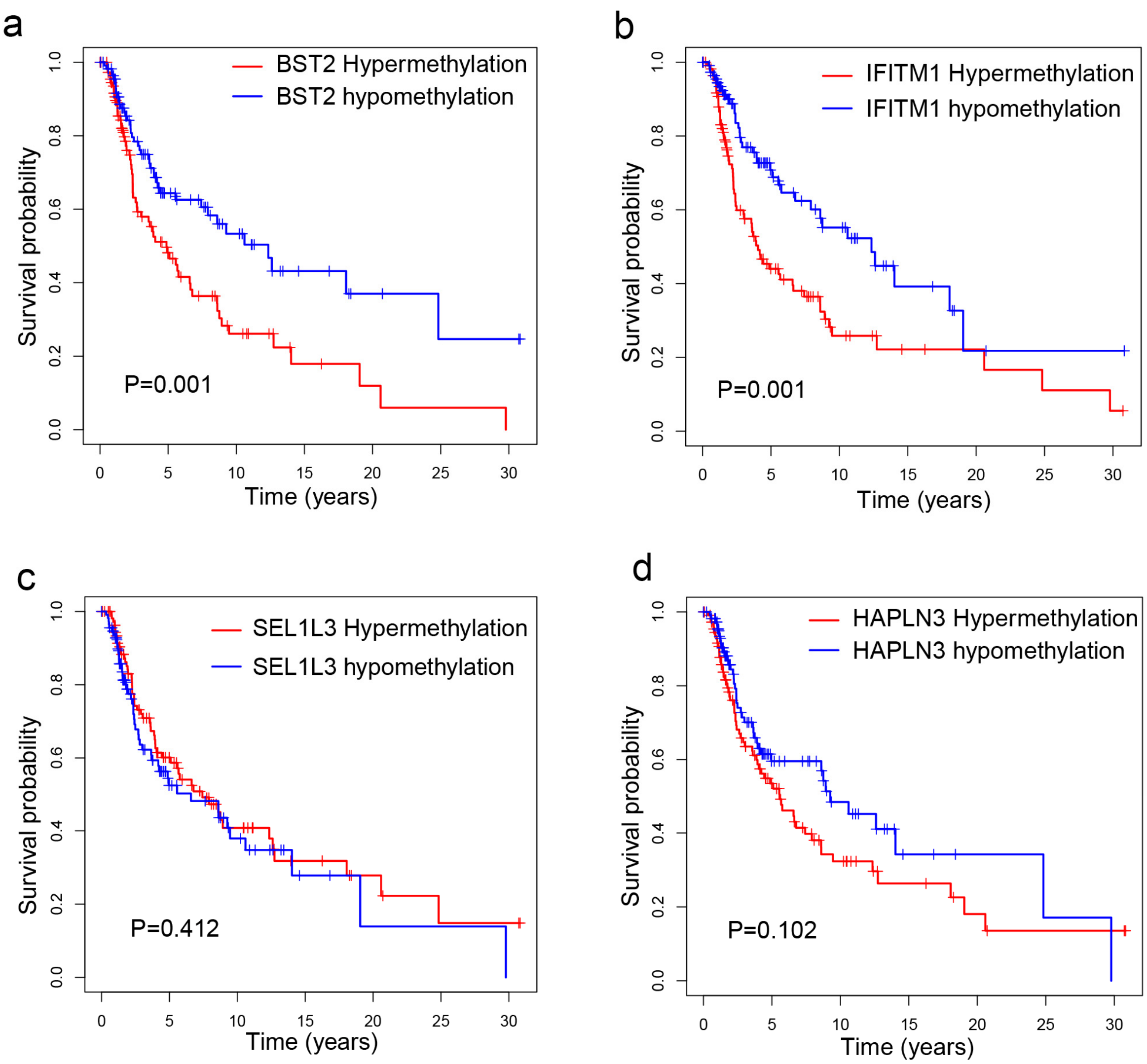

Supplementary Fig. 6. Correlations of *SEL1L3*, *HAPLN3*, *BST2,* and *IFITM1* gene methylation levels with overall survival.

(a-d) Kaplan-Meier curves of the survival rate of patients with high and low methylation (the cutoff value is the median) level of *BST2* **(a)**, *IFITM1* **(b)**, *SEL1L3* **(c)**, and *HAPLN3* **(d)**. Statistical significance was determined by the log-rank test.
