# Supplementary material for: A four-gene signature predicts survival and anti-CTLA4 immunotherapeutic responses based on immune classification of melanoma
Source: Commun Biol. 2021 Mar 22;4:383. doi: 10.1038/s42003-021-01911-x (PMC7985195; doi:10.1038/s42003-021-01911-x)
Supplement: Supplementary file 2 — Description of Additional Supplementary Files [file 42003_2021_1911_MOESM2_ESM.pdf]

Description of Additional Supplementary Files

File name: Supplementary Data 1

Description: 28 immune-associated gene sets.

File name: Supplementary Data 2

Description: Differentially expressed genes between L and H subtypes.

File name: Supplementary Data 3

Description: Source data for Figures 1-6.
