# Supplementary material for: A four-gene signature predicts survival and anti-CTLA4 immunotherapeutic responses based on immune classification of melanoma
Source: Commun Biol. 2021 Mar 22;4:383. doi: 10.1038/s42003-021-01911-x (PMC7985195; doi:10.1038/s42003-021-01911-x)
Supplement: Supplementary file 6 — Reporting Summary [file 42003_2021_1911_MOESM6_ESM.pdf]

nature research

Corresponding author(s): Li Ma

Last updated by author(s): Feb 13, 2021

Reporting Summary

Nature Research wishes to improve the reproducibility of the work that we publish. This form provides structure for consistency and transparency in reporting. For further information on Nature Research policies, see our Editorial Policies and the Editorial Policy Checklist.

Statistics

For all statistical analyses, confirm that the following items are present in the figure legend, table legend, main text, or Methods section.

n/a	Confirmed	
☐	☒	The exact sample size (n) for each experimental group/condition, given as a discrete number and unit of measurement
☐	☒	A statement on whether measurements were taken from distinct samples or whether the same sample was measured repeatedly
☐	☒	The statistical test(s) used AND whether they are one- or two-sided<br>*Only common tests should be described solely by name; describe more complex techniques in the Methods section.*
☒	☐	A description of all covariates tested
☒	☐	A description of any assumptions or corrections, such as tests of normality and adjustment for multiple comparisons
☐	☒	A full description of the statistical parameters including central tendency (e.g. means) or other basic estimates (e.g. regression coefficient) AND variation (e.g. standard deviation) or associated estimates of uncertainty (e.g. confidence intervals)
☐	☒	For null hypothesis testing, the test statistic (e.g. F, t, r) with confidence intervals, effect sizes, degrees of freedom and P value noted<br>*Give P values as exact values whenever suitable.*
☒	☐	For Bayesian analysis, information on the choice of priors and Markov chain Monte Carlo settings
☐	☒	For hierarchical and complex designs, identification of the appropriate level for tests and full reporting of outcomes
☐	☒	Estimates of effect sizes (e.g. Cohen's d, Pearson's r), indicating how they were calculated

Our web collection on statistics for biologists contains articles on many of the points above.

Software and code

Policy information about availability of computer code

Data collection	Data used in this research were downloaded from the links below :<br>TCGA (https://portal.gdc.cancer.gov), melanoma dataset; Gene Expression Omnibus (https://www.ncbi.nlm.nih.gov/gds/), accession number: GSE65904; and dbGap (https://www.ncbi.nlm.nih.gov/gap/), accession number: phs000452.v2.p1.
Data analysis	We performed data analyses by using R (version 3.6.1)

For manuscripts utilizing custom algorithms or software that are central to the research but not yet described in published literature, software must be made available to editors and reviewers. We strongly encourage code deposition in a community repository (e.g. GitHub). See the Nature Research guidelines for submitting code & software for further information.

Data

Policy information about availability of data

All manuscripts must include a data availability statement. This statement should provide the following information, where applicable:

- Accession codes, unique identifiers, or web links for publicly available datasets
- A list of figures that have associated raw data
- A description of any restrictions on data availability

The fragments per kilobase of transcript per million mapped reads (FPKM) values of the SKCM RNA-sequencing data and corresponding clinical data were downloaded from TCGA (https://portal.gdc.cancer.gov), and the sample size is 471. The accession number of the dataset from Gene Expression Omnibus (GEO; https://www.ncbi.nlm.nih.gov/gds/) is GSE65904, and the sample size is 214. The accession number of the dataset from dbGap (https://www.ncbi.nlm.nih.gov/gap/) is phs000452.v2.p1, and the sample size is 42.

Field-specific reporting

Please select the one below that is the best fit for your research. If you are not sure, read the appropriate sections before making your selection.

☒ Life sciences ☐ Behavioural & social sciences ☐ Ecological, evolutionary & environmental sciences

For a reference copy of the document with all sections, see nature.com/documents/nr-reporting-summary-flat.pdf

Life sciences study design

All studies must disclose on these points even when the disclosure is negative.

Sample size	The training dataset downloaded from TCGA contains 471 samples. The validation dataset downloaded from GEO contains 214 samples . The dataset with ipilimumab therapy response information available (downloaded from dbGap) contains 42 samples.
Data exclusions	All samples with RNA-seq data and survival data were included in this study.
Replication	Not applicable.
Randomization	Not applicable.
Blinding	Not applicable.

Reporting for specific materials, systems and methods

We require information from authors about some types of materials, experimental systems and methods used in many studies. Here, indicate whether each material, system or method listed is relevant to your study. If you are not sure if a list item applies to your research, read the appropriate section before selecting a response.

Materials & experimental systems

n/a	Involved in the study	
☒	☐	Antibodies
☒	☐	Eukaryotic cell lines
☒	☐	Palaeontology and archaeology
☒	☐	Animals and other organisms
☒	☐	Human research participants
☒	☐	Clinical data
☒	☐	Dual use research of concern

Methods

n/a	Involved in the study	
☒	☐	ChIP-seq
☒	☐	Flow cytometry
☒	☐	MRI-based neuroimaging
